# Damage-associated molecular patterns in bacteraemic infection, including a comparative analysis with bacterial DNA, a pathogen-associated molecular pattern

**DOI:** 10.1038/s41598-024-74868-6

**Published:** 2024-10-08

**Authors:** Helena Alpkvist, Ingrid Ziegler, Paula Mölling, Elisabet Tina, Linnea Sellvén, Anna Norrby-Teglund, Sara Cajander, Kristoffer Strålin

**Affiliations:** 1 Department of Medicine Huddinge, Unit of Infectious Diseases, Karolinska Institutet, Stockholm, Sweden; 2 Department of Infectious Diseases, I73 Karolinska University Hospital, 141 86 Stockholm, Sweden; 3 Department of Infectious Diseases, Södersjukhuset, Stockholm, Sweden; 4 Department of Laboratory Medicine, Faculty of Medicine and Health, Örebro University, Örebro, Sweden; 5 Department of Clinical Research Centre, Faculty of Medicine and Health, Örebro University, Örebro, Sweden; 6 Department of Infectious Diseases, Örebro University Hospital, Faculty of Medicine and Health, Örebro University, Örebro, Sweden; 7 Center for Infectious Medicine, Department of Medicine Huddinge, Karolinska Institutet, Stockholm, Sweden

**Keywords:** Damage-associated molecular patterns, DAMP, Pathogen-associated molecular patterns, PAMP, Bacteraemic infection, Bacteraemia, Sepsis, Nuclear DNA, nDNA, HLA-DR, Biomarkers, Medical research

## Abstract

Damage-associated molecular patterns (DAMPs) and pathogen-associated molecular patterns (PAMPs) are key triggers of inflammation in sepsis. However, they have rarely been studied simultaneously. Thus, in the present study of patients with bacteraemic infection, we aimed to study how DAMP dynamics are linked to disease severity and outcome and to compare diagnostic and prognostic properties of a DAMP and a previously analysed PAMP (16S rDNA). In a prospective study of adult patients hospitalized with culture-proven community-onset bacteraemic infection, caused by *Streptococcus pneumonia* (n = 30), *Staphylococcus aureus* (n = 27), or *Escherichia coli* (n = 26), dynamics of a PAMP, i.e. 16S rDNA, have previously been presented. For the present study, blood samples obtained on hospital days 1–2 (when blood culture was positive), 3–4, 7 ± 1, 14 ± 2, and 28 ± 4 were analysed for four different DAMPs, i.e., nuclear DNA (nDNA), mitochondrial DNA (mtDNA), heat shock protein 90 alpha (HSP90α), and extracellular high mobility group box 1 (HMGB1). Sepsis was defined according to the Sepsis-3 criteria. The study outcomes were sepsis at admission and negative outcome, defined as intensive care unit (ICU) admission and/or death within 60 days. Of 83 study patients, sepsis was noted in 41 patients (49%) and a negative outcome was noted in 17 patients (20%). nDNA had areas under the receiver operating characteristic (ROC) curves of 0.78 for sepsis and 0.76 for negative outcome, which were higher than those of the other DAMPs and additional biomarkers (CRP, IL-6, IL-8, and IL-10). The nDNA and positive 16S rDNA results on day 1–2 were correlated with each other (r = 0.68, *p* < 0.001). Multivariate analyses showed that high day 1–2 concentrations of both nDNA and 16S rDNA were independently associated with sepsis. In addition, high day 1–2 concentration of nDNA was independently associated with negative outcomes. While 16S rDNA dissipated from the circulation within days, nDNA concentrations remained elevated throughout the follow-up period in patients with negative outcome. In conclusion, nDNA outperformed the other DAMPs regarding sepsis detection and outcome prediction. Both nDNA (a DAMP) and 16S rDNA (a PAMP) were independently linked to sepsis; nDNA was also associated with negative outcomes and persisted elevated in such cases. This highlights nDNA as an interesting marker within sepsis pathogenesis and as a promising clinical biomarker, warranting further studies.

## Background

Bacteraemic infections, marked by the presence of bacteria in the bloodstream, pose a significant threat to public health. The outcome of bacteraemic infection vary widely, some patients respond swiftly to treatment, while others develop sepsis with life-threatening organ dysfunction^1,2^. Understanding the intricate molecular mechanisms involved in the host response to such infections is crucial for improving diagnostic and therapeutic approaches. Moreover, new biomarkers are needed for screening, risk assessment, and monitoring treatment response in patients with sepsis^3,4^.

Damage-associated molecular patterns (DAMPs), which originate from host cells undergoing damage or necrosis, activate the innate immune cells via pattern recognition receptors (PRRs) and play key roles in the intricate immune response to infections. High bloodstream concentrations of various DAMPs have been correlated with negative outcome in patients with severe bacterial infections^5–8^.

PRRs are also triggered by pathogen-associated molecular patterns (PAMPs), which are microbial components, e.g. bacterial DNA^9^. Systemic^10–12^ and local concentrations^13,14^ of bacterial DNA have been linked to severity of bacterial infections. However, the interplay between DAMPs and PAMPs, and their roles as initiators of inflammation during bacterial infections, remain unclear, as DAMP and PAMP concentrations have rarely been studied in the same cohorts of patients.

In this study of patients with bacteraemic infection caused by *Streptococcus pneumoniae*, *Staphylococcus aureus*, and *Escherichia coli*, we aimed to investigate the dynamics of four DAMPs: nuclear DNA (nDNA), mitochondrial DNA (mtDNA), high mobility group box 1 (HMGB1), and heat shock protein 90 alpha (HSP90α), in relation to the presence of sepsis and outcome. Additionally, we sought to compare DAMP results to the results of a PAMP, i.e. bacterial DNA (16S rDNA), which have already been presented^15^. Through this exploration, we aimed to deepen our understanding on the pathogenesis of bacteraemic infections, potentially leading to enhanced diagnostic and prognostic capabilities.

## Methods

### Patients

This prospective study of patients with community-onset bacteraemic infection was conducted at Örebro University Hospital, Sweden, from 2011 to 2014^15,16^. As we have previously reported^15^, the study included 83 patients with community-onset bacteraemic infection caused by *S. pneumonia* (n = 30), *S. aureus* (n = 27), or *E. coli* (n = 26). Patients were enrolled in the study when blood cultures (BCs) demonstrated growth of S. *pneumoniae*, *S. aureus*, or *E. coli* within 48 h of hospital admission. The study also included 31 healthy controls, who were randomly collected blood donors ≥ 40 years old.

### Clinical data and severity classification

Clinical data regarding demographics, comorbidities, length of hospital stay, intensive care unit (ICU) admission and mortality were obtained from patient records. Comorbidity was assessed using the Charlson comorbidity index (CCI)^17^.

Neutropenia (neutrophil count < 0.5 × 109/L) or immunosuppressive medication (methotrexate, chemotherapeutics, or cortisol dosing equivalent to ≥ 20 mg prednisolone) was considered immunosuppression prior to infection.

Acute disease severity was assessed according to the Sepsis-3 criteria, and patients with an acute sequential organ failure assessment (SOFA) score increase of ≥ 2 were considered to have sepsis^18^.

A negative outcome was defined as admission to an ICU and/or death within 60 days after admission.

### Blood samples

Two pairs of BCs were collected from each patient on hospital admission and were analysed according to routine practice.

EDTA and serum blood samples were collected on days 1–2 (n = 73), 3–4 (n = 55), 7 ± 1 (n = 67), 14 ± 2 (n = 58), and 28 ± 4 (n = 53) after hospital admission.

From the EDTA blood samples, 1 mL whole blood was immediately analysed with flow-cytometry for HLA-DR, 1 mL whole blood was preserved for 16S rDNA PCR analysis, and the remaining blood was centrifuged to obtain plasma that was frozen in aliquots at − 80 °C. The serum samples were centrifuged to obtain serum that was frozen in aliquots at − 80 °C.

On the days of the study samples, routine sampling for CRP, neutrophil count, and lymphocyte count were also performed.

### nDNA and mtDNA

The qPCR analysis of nDNA and mtDNA in plasma samples were based on previous studies by Nakahira et al.^19^ and Timmermanns et al.^7^. In brief, mtDNA was identified using the NADH gene, while nDNA was detected through the GAPDH gene. To determine quantity of copies/PCR, standard curves were constructed (101–106 copies/PCR in TE-buffer; pH 8.0; Thermo Fischer Scientific Inc., Walton, MA, USA) using plasmids containing the gene sequence for NADH (MTND1) and GAPDH, respectively (Origene Technologies Inc., Rockville, MA, USA). The qPCR reactions were performed in duplicates in a total volume of 20 µL containing 2 µL sample or standard plasmid and 18 µL mastermix (Power SYBR™ Green PCR Master Mix (Applied Biosystems™, Thermo Fischer Scientific), 300 nM primers (Sigma Aldrich Company Limited, England) and nuclease free water) using 96-well fast plates (Applied Biosystems™). For qPCR amplification of samples, a QuantStudio™ 7 (QS7) real time PCR system (Applied Biosystems™) was used, and the plates were run in the PCR program; + 50 °C pre incubation, then + 95 °C denaturation, and then + 60 °C annealing and extension, repeated for 40 cycles. The qPCR results were then calculated using the QS7 v1.3 software with automatic baseline threshold setting, generating standard curves for each gene and plate.

### HSP90α and HMGB1

The ELISA assays for HSP90α (Enzo Life Sciences, Lausen, Switzerland) and HMGB1 (IBL international, Hamburg, Germany) were used on serum, according to the manufacturer’s instructions to assess serum concentrations. For HSP90α a standard curve range of 0.0625–4 ng/mL was used, and for HMGB1 2–80 ng/mL. Samples were analysed in duplicates.

### Bacterial 16S rDNA

The diagnostic procedures, analysis with droplet digital PCR for 16S rDNA on whole blood, and results of the study subjects in the present study have already been reported^15^.

### Interleukins

Plasma concentrations of IL1β, IL 6, IL 8, IL10 and TNF-α were simultaneous determined using MILLIPLEX® MAP Human High Sensitivity T Cell Panel (EMD Millipore Corporation, Billerica, MA, USA). The assay was performed on undiluted samples in duplicates according to the manufacturers’ protocol. Measurements and analyses were performed using a Luminex 200™ (Luminex Corporation, Austin, TX, USA) and xPONENT® software v 3.1 (Luminex).

### Monocytic HLA-DR expression

The diagnostic procedures, analysis with flow cytometry for monocytic HLA-DR (mHLA-DR) on EDTA whole blood, and results of the study subjects in the present study, have already been reported^16^.

### Statistical analysis

The Mann–Whitney U test was used for comparisons between two different groups, and the Kruskal Wallis test was used for comparisons between more than two groups. The Spearman correlation was used to evaluate of the relationship between two variables. Linear and binary logistic regression analyses, univariate and multivariate, was performed. To enable log transformation of 16S rDNA data, where many patients exhibited 0 copies/ml, 0.01 copies/ml was added to each patient value prior to log transformation.

The SPSS software package (IBM, version 28) was used for all statistical analyses. A *p*-value of < 0.05 was considered significant.

In the design of figures, a small number of outliers were omitted from the visual presentations to allow for the use of a linear scale while maintaining readability. However, all outliers were included in all statistical analyses.

## Results

### Study cohort and controls

The 83 study patients are described in Supplementary Table S1. Characteristics of the three subgroups with different microbiological aetiology have been presented in our previous article^15^. The median age was 72 years, 47% were female. Sepsis on admission was observed in 41 patients (49%), with 25 patients having a SOFA score of 2–3 and 16 patients having a SOFA score of ≥ 4. A negative outcome was observed in 17 patients (20%), 12 of whom were admitted to the ICU and 7 of whom died within 60 days. The 31 healthy controls (blood donors) had a median age of 54 years, 23% were female.

### DAMPs in patients and controls

Supplementary Fig. S1 shows the median DAMP concentrations over time. DAMP concentrations were significantly higher in patients than in controls during the study period, except for HMGB1. HMGB1 concentrations in patients decreased to concentrations comparable to those in healthy controls in the latter half of the study period.

The DAMP concentrations on day 1–2 of patients with different aetiologies are shown in Supplementary Fig. S2. The nDNA concentrations on day 1–2 were significantly higher for the patients with *S. pneumoniae* bacteraemia (median 534 copies/μL, interquartile range 373–1157, copies/μL) and *S. aureus* bacteraemia (median 661 copies/μL, interquartile range 301–1068, copies/μL) than for those with *E. coli* bacteraemia (median 215 copies/μL, interquartile range 149–316, copies/μL), p < 0.001 for both comparisons. There were no differences in median day 1–2 concentrations of mtDNA, HSP90α or HMGB1between patients with different aetiologies.

### DAMP dynamics and disease severity

**Fig. 1:**
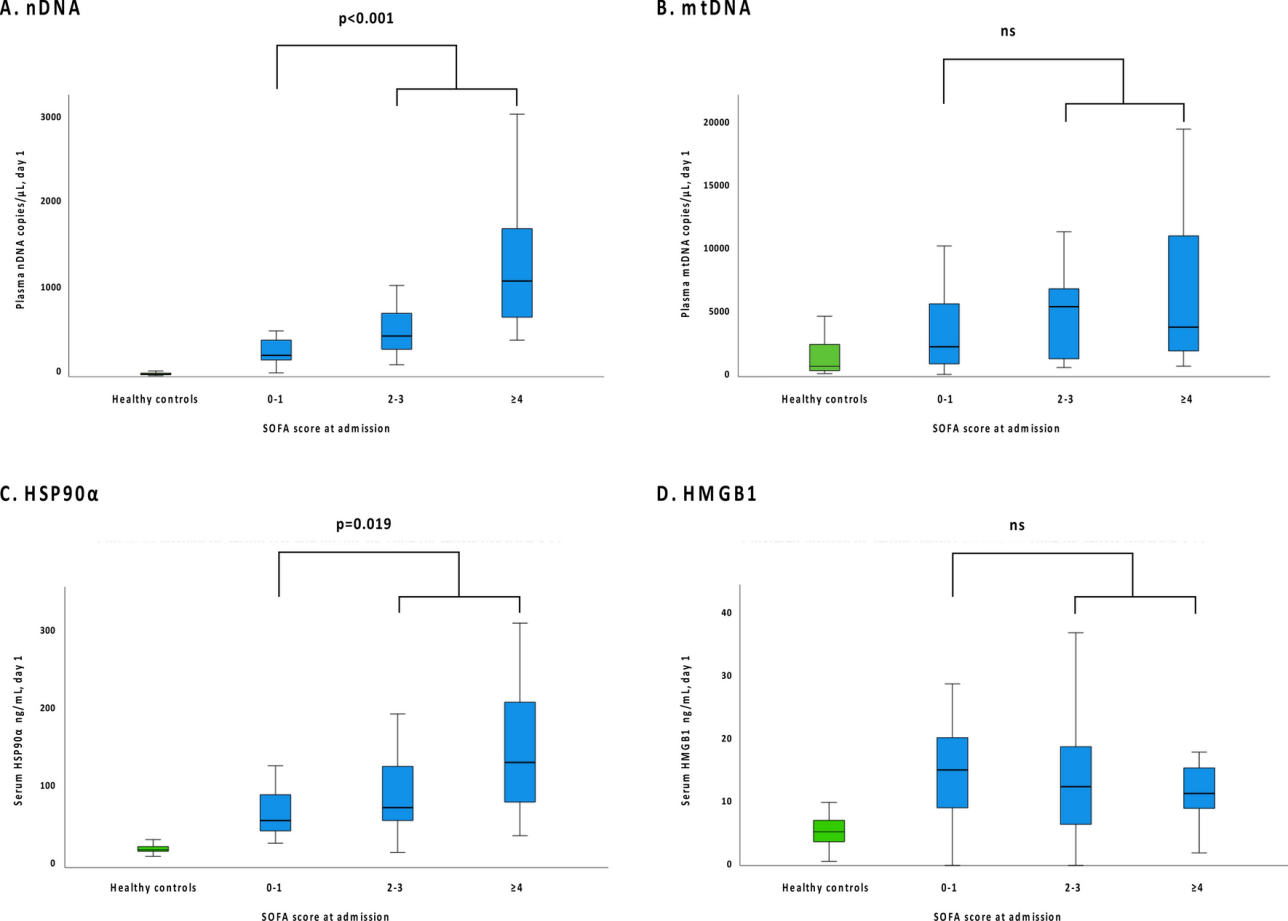
**(A–D)** Plasma-concentrations of the DAMPs nDNA (**A**) and mtDNA (**B**), and serum-concentrations of HSP90α (**C**) and HMGB1(D) at admission, related to admission SOFA score. A comparison between patients with SOFA score ≥ 2 (sepsis) and SOFA score < 2 (not sepsis) is included. A *p*-value of < 0.05 was considered significant, ns = non-significant. A small number of outliers were omitted from the visual presentations to allow for the use of a linear scale while maintaining readability. However, all outliers were included in all statistical analyses.

As noted in Fig. 1, patients with sepsis at admission had significantly greater day 1–2 concentrations of nDNA and HSP90α than patients without sepsis. However, the concentrations of mtDNA and HMGB1 did not differ between those with or without sepsis. On day 1–2, the nDNA and HSP90α concentrations were correlated with the admission SOFA score (r = 0.56, *p* < 0.001 and r = 0.34, *p* = 0.003, respectively).

**Fig. 2:**
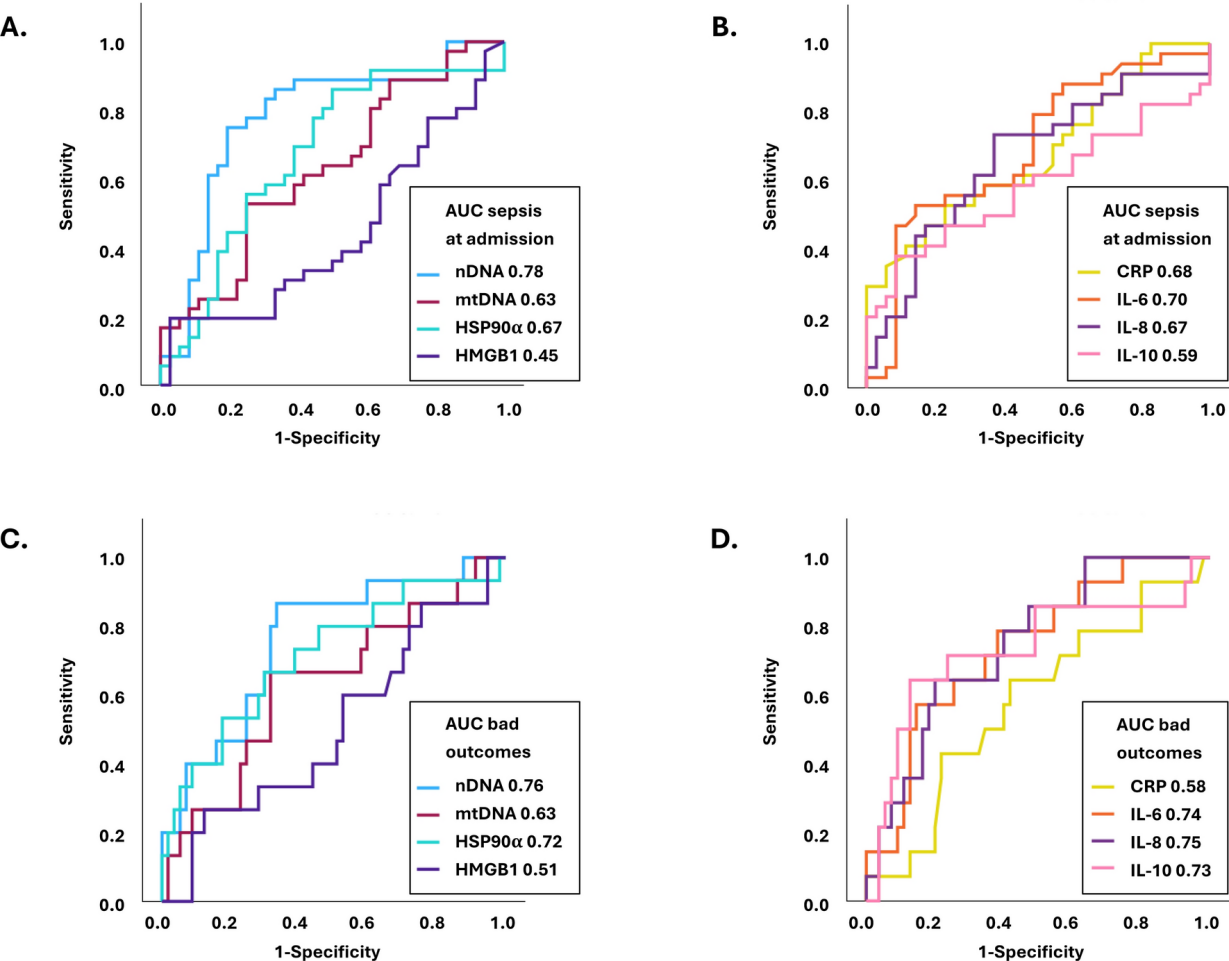
**(A**–**D)** Receiver operating characteristic curve analyses of day 1–2 concentrations of four DAMPS, i.e. plasma nDNA copies/μL, plasma mtDNA copies/μL, serum HSP90α ng/mL and serum HMGB1 ng/mL, for detection of sepsis at admission (**A**) and prediction of negative outcome (**C**). Comparison with CRP, IL-6, IL-8, and IL-10 (**B** and **D**). AUC, area under the curve.

Figure 2 shows receiver operating characteristic (ROC) curve analyses on the performances of DAMPs and inflammatory markers on day 1–2 for detecting sepsis and for anticipating a subsequent negative outcome. For detecting sepsis, the areas under the ROC curves were 0.78 for nDNA, 0.63 for mtDNA, 0.67 for HSP90α, and 0.45 for HMGB1, and 0.59–0.70 for inflammatory markers (CRP, IL6, IL-8, and IL-10). For predicting negative outcome, the areas under the ROC curves were 0.76 for nDNA, 0.63 for mtDNA, 0.72 for HSP90α, and 0.51 for HMGB1, 0.73–0.75 for the interleukins, and 0.58 for CRP.

**Fig. 3:**
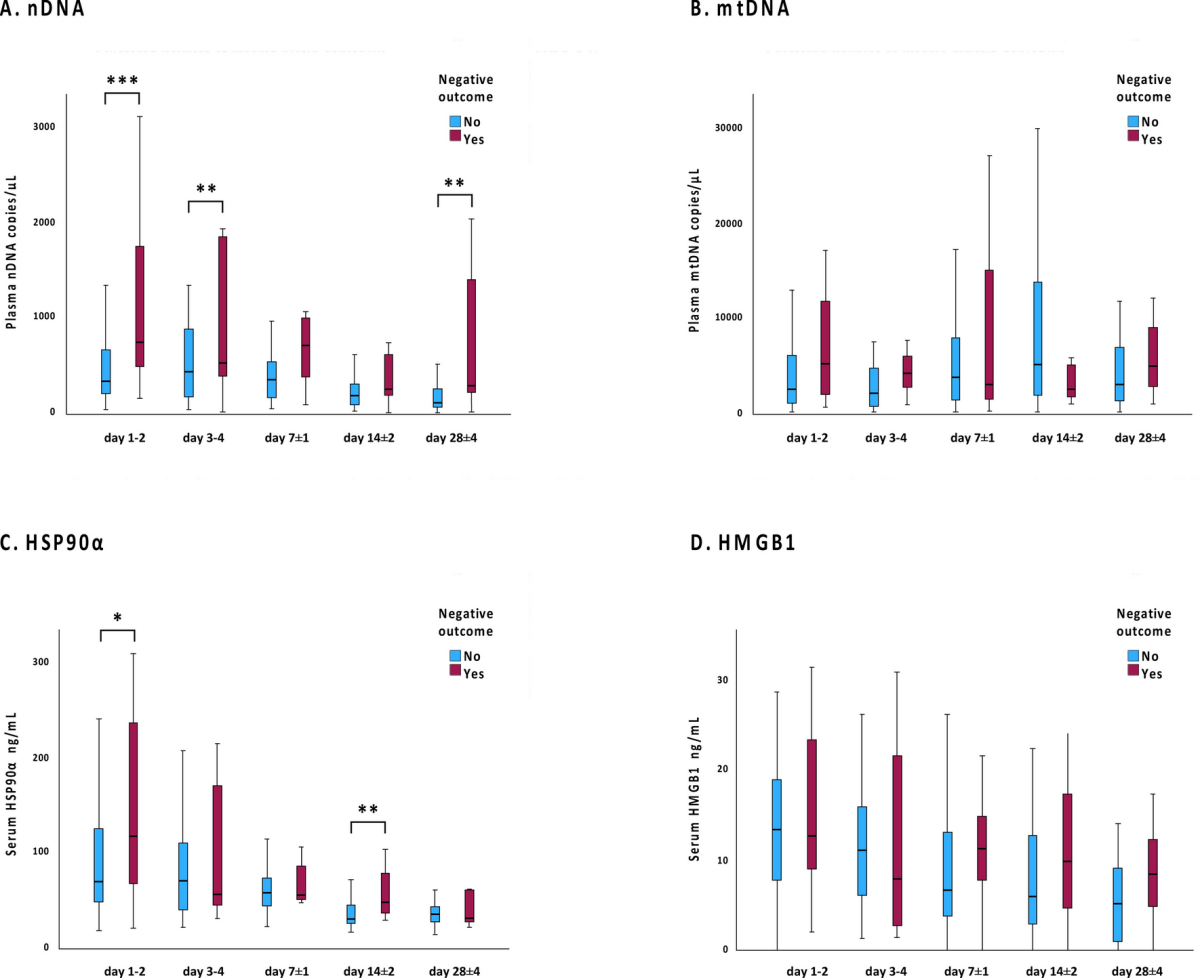
**(A**–**D)** DAMP concentrations day 1 to day 28 in patients with and without negative outcome (admission to an ICU and/or death within 60 days after admission). * = *p* < 0.05, ** = *p* < 0.01 and *** = *p* < 0.001. A small number of outliers were omitted from the visual presentations to allow for the use of a linear scale while maintaining readability. However, all outliers were included in all statistical analyses.

Regarding dynamics of DAMPs, patients with sepsis had significantly higher nDNA concentration than those without sepsis from day 1–2 to day 14 ± 2, but significantly higher HSP90α concentration only on day 1–2, see supplementary Fig. S3. In addition, patients with a negative outcome demonstrated significantly higher nDNA concentration than those without even on day 28 ± 4, see Fig. 3.

As shown in Supplementary Fig. S3 and Fig. 3 no correlation was found between the concentrations of mtDNA or HMGB1 and sepsis or a negative outcome, at any point during the 28-day study period.

### Selection of nDNA as DAMP in remaining analyses

Given that nDNA, among the four DAMPs studied, demonstrated the strongest correlation with admission SOFA score and exhibited the highest performance in detecting sepsis and predicting negative outcomes, it was selected as the representative DAMP for use in the subsequent analyses of the study.

### nDNA related to markers of inflammation and immunosuppression

Table 1 shows correlations between nDNA and established biomarkers for inflammation and immunosuppression, throughout the 28-day study period. nDNA concentrations were generally positively correlated to CRP, neutrophil count, and IL6, IL8 and TNFα concentrations, and negatively correlated to lymphocyte count and mHLA-DR.

**Table 1:** Correlation between nDNA and biomarkers for inflammation and immunosuppression, from day 1 to day 28.

		nDNA	
	Day	r	*p*
Inflammation			
CRP	1	0.52	< 0.001
	3	0.55	< 0.001
	7	0.52	< 0.001
	14	0.33	0.025
	28	0.62	< 0.001
Neutrophil count	1	ns	ns
	3	0.58	< 0.001
	7	0.60	< 0.001
	14	0.45	< 0.001
	28	0.39	0.004
IL6	1	0.58	< 0.001
	3	0.38	0.005
	7	ns	ns
	14	ns	ns
	28	0.38	0.009
IL8	1	0.58	< 0.001
	3	0.34	0.011
	7	0.43	< 0.001
	14	0.38	0.004
	28	0.57	< 0.001
TNFα	1	0.28	0.016
	3	0.34	0.013
	7	0.30	0.015
	14	ns	ns
	28	ns	ns
IL1β	1	ns	ns
	3	ns	ns
	7	ns	ns
	14	ns	ns
	28	ns	ns
Immunosuppression			
Lymphocyte count	1	− 0.25	0.037
	3	ns	ns
	7	ns	ns
	14	− 0.32	0.017
	28	− 0.32	0.021
mHLA-DR	1	− 0.35	0.004
	3	− 0.53	< 0.001
	7	− 0.46	< 0.001
	14	− 0.27	0.046
	28	− 0.33	0.017
IL10	1	0.35	0.003
	3	0.33	0.015
	7	ns	ns
	14	ns	ns
	28	ns	ns

The nonparametric Spearman correlation was used to evaluate of the relationship between two variables. A *p*-value of < 0.05 was considered significant, ns = non-significant.

### nDNA in relation to patient factors

As shown in Supplementary Table S2, no significant correlations were observed between day 1–2 nDNA concentrations and baseline patient characteristics, including gender, age, or the presence of comorbidity as assessed by the CCI. The correlation between nDNA and specific comorbidities was not studied.

### DAMP (nDNA) versus PAMP (16S rDNA)

The selected DAMP, nDNA, was then compared with a PAMP, 16S rDNA, for which the results have already been presented^15^. Among 63 patients tested for 16S rDNA, 33 had detectable bacterial 16S rDNA on day 1–2, with no significant differences in concentrations between patients with different aetiologies^15^.

**Fig. 4:**
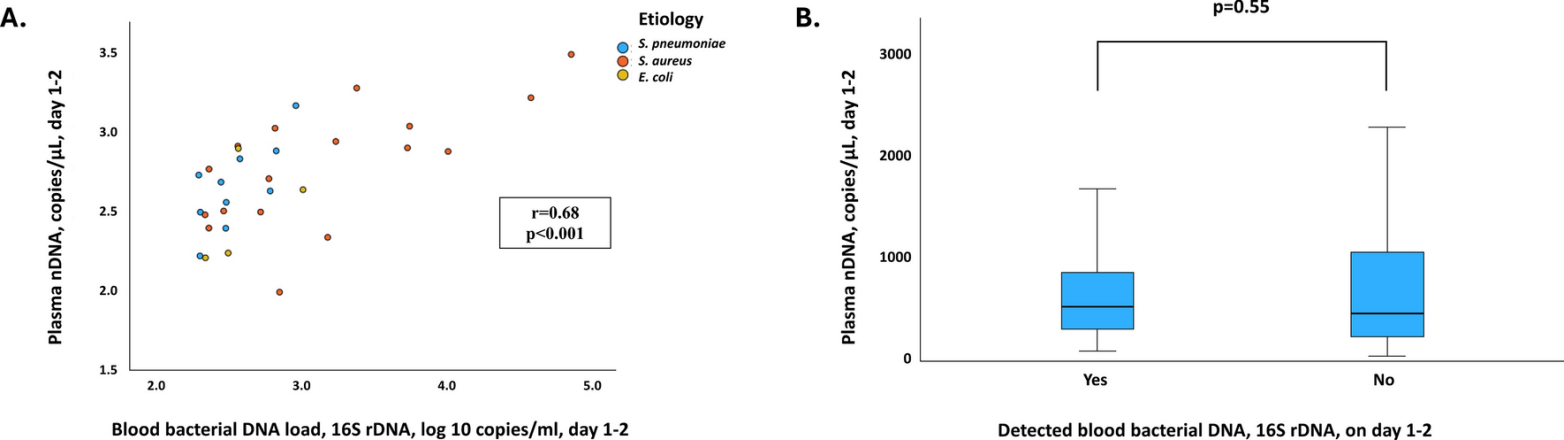
**(A**–**B)** (**A**) Correlation between day 1–2 concentrations of plasma nDNA and blood 16S rDNA, in 33 patients with detected 16S rDNA. Spearman correlation was used. (**B**) Day 1–2 concentrations of nDNA, in patients with detected bacterial DNA (median 529, range 98–3028 copies/μL) versus patients with no detected bacterial DNA (median 462, range 44–3088 copies/μL).

Among patients with detected 16S rDNA, there was a good correlation between 16S rDNA and nDNA concentrations on day 1–2 (r = 0.68 and *p* < 0.001), see Fig. 4A. As noted visually, the correlation appeared to be present within the three aetiological subgroups.

Notably, nDNA concentrations on day 1–2 did not differ between patients with or without detectable 16S rDNA, Fig. 4B.

Next, we explored whether nDNA and 16S rDNA concentrations were linked to the presence of sepsis. Multivariate analysis, as shown in Table 2, included day 1–2 concentrations of nDNA and 16S rDNA, along with factors known to influence the risk of sepsis, such as age, gender, and the presence of comorbidity. We found that both high concentrations of nDNA and high concentrations of 16S rDNA were independently associated with sepsis at admission. The same was true for male gender.

**Table 2:** Odds ratios (OR) with confidence intervals (CI) for sepsis at admission (SOFA score ≥ 2), in 63 patients with bacteraemic infection.

Factors	Odds ratio (95% CI)	*p*-value
Male gender	5.89 (1.47–23.67)	0.012
Age	1.03 (0.98–1.09)	0.20
Charlson comorbidity score ≥ 1	1.33 (0.36–4.87)	0.67
Plasma nDNA, log10 copies/μL (DAMP) on day 1–2	12.53 (1.92–81.54)	0.008
Plasma 16S DNA, log10 copies/ml (PAMP) on day 1–2	1.34 (1.02–1.75)	0.033

Binary logistic regression, multivariate analysis.

Subsequently, we investigated whether concentrations of nDNA and 16S rDNA on day 1–2 were associated with a negative outcome, as detailed in Table 3. We included known risk factors for negative outcome, such as age, gender, comorbidity, and the presence of sepsis at admission. We found that high concentration of nDNA was independently associated with a negative outcome, as were male gender and the presence of comorbidity according to the CCI. High concentrations of 16S rDNA showed borderline significance (*p*-value 0.053). However, sepsis at admission was not independently associated with a negative outcome.

**Table 3:** Odds ratios (OR) with confidence intervals (CI) for negative outcome (admission to an intensive care unit and/or death within 60 days), in 63 patients with bacteraemic infection.

Factors	Odds ratio (95% CI)	*p*-value
Male gender	7.00 (0.99–49.31)	0.051
Age	1.06 (0.97–1.16)	0.21
Charlson comorbidity score ≥ 1	32.50 (1.90–555.99)	0.016
Sepsis at admission (SOFA score ≥ 2)	1.73 (0.20–15.13)	0.62
Plasma nDNA, log10 copies/μL (DAMP) on day 1–2	17.31 (1.10–271.40)	0.042
Plasma 16S DNA, log10 copies/ml (PAMP) on day 1–2	1.41 (0.99–2.00)	0.053

Binary logistic regression, multivariate analysis.

The dynamics of nDNA and 16S rDNA differed significantly. As previously reported, 16S rDNA levels rapidly declined, with detection in only three patients on day 14 ± 2 and none on day 28 ± 4^15^. In contrast, elevated nDNA concentrations were observed throughout the study period (Supplementary Fig. S1), and by the end of the 28-day study, nDNA concentrations were significantly higher in patients with a negative outcome compared to the rest of the cohort (Fig. 3).

## Discussion

This study on the dynamics of DAMPs in adult patients with bacteraemic infection showed that nDNA outperformed other DAMPs in detecting sepsis and predicting negative outcome. Comparative analysis of a DAMP (nDNA) and a PAMP (16S rDNA) revealed a correlation between these markers; however, both exhibited an independent correlation with sepsis. Additionally, nDNA was correlated with negative outcomes, and nDNA concentrations remained persistently elevated in these patients.

In the first part of the study, we aimed to evaluate the performance and dynamics of four different DAMPs. Few studies have investigated the dynamics of DAMPs during bacterial infections. Timmerman et al.^7^ reported consistent elevations in both nDNA and mtDNA from day 1–2 to 28 ± 4 in septic shock patients compared to healthy controls. In our study, we observed a similar trend in which all four DAMPs were elevated in patients relative to controls.

Based on its association with clinical severity, nDNA was identified as the best performing DAMP. High concentration of nDNA was linked to severe disease. Specifically, the day 1–2 concentration of nDNA was correlated with the degree of organ failure, as assessed by the SOFA score. This observation is consistent with previous sepsis research linking elevated nDNA concentrations to renal failure^20^ and increased mortality^5^. In the present study, the link to severe disease was reinforced by a robust and consistent correlation between nDNA concentrations and results of biomarkers of inflammation and immunosuppression.

Interestingly, our study showed that patients with negative outcome had significantly higher nDNA concentrations by the end of the 28-day study period than did the rest of the cohort. This observation is in line with murine sepsis models in which therapeutic reduction of cell-free DNA led to improved outcome^21,22^, supporting the role of nDNA in sepsis pathogenesis, and suggesting a potential of targeted interventions in patients with persistently high concentrations of nDNA. Taken together, these data strongly support further studies of the role of nDNA within sepsis pathogenesis.

nDNA and 16S rDNA concentrations were strongly correlated in the day 1–2 samples. One possible explanation for this finding could be that nDNA is released into the circulation from organs affected by the infection, and that the 16S rDNA concentration reflects the extent of the infection. There could also be a more direct link between the PAMP and DAMP, as Timmerman et al.^7^, found an increase in systemic nDNA concentrations in healthy controls after administration of LPS, a PAMP. However, in addition to the correlation between nDNA and 16S rDNA among patients with detected 16S rDNA, the present study showed a lack of nDNA difference between patients with and without detected 16S rDNA. Most likely, this illustrates problems with false-negative 16S rDNA results. These data and the low positivity rate reduce the usefulness of the 16S rDNA method. However, the independent correlation of 16S rDNA with sepsis motivates efforts to improve the 16S rDNA method and enhance sensitivity.

The independent correlations of nDNA with sepsis and negative outcome underscore the diagnostic and prognostic potentials of this biomarker, warranting studies on broader cohorts of unselected patients with bacterial infections.

The present study did not show any correlation between nDNA concentrations and baseline factors such as gender, age, or CCI. This lack of correlation suggests that nDNA primarily reflects disease severity, without being confounded by baseline patient characteristics.

The study has limitations. First, the sample size was relatively small, and the data were derived from a single cohort. The sample size limited the possibilities to include an optimal outcome measure for negative outcome, as only 7 patients died within 60 days. Thus, to enable an analysis of the clinical course, we chose to use the combined outcome measure of ICU admission and/or death to define a negative outcome. Second, due to the inclusion criteria of positive blood culture, admission samples were not used in this study. Thus, rapid dynamic changes within the first day of hospitalisation could not be detected with our study design. Third, even though this was a prospective study, many patients had missing samples at several sample occasions. A major reason was difficulties to obtain samples after discharge. Forth, as the study specifically focused on patients with bacteraemic infection, the results may not be generalizable to a broader, unselected sepsis population.

Among the strengths of this study is the use of a well-defined cohort, consisting entirely of patients with confirmed bacteraemic infection. The study features a clear and methodical design, with blood samples collected at specified intervals throughout the study duration. Additionally, the simultaneous study of DAMPs and PAMPs within the same cohort enhances our understanding of their interplay in bacteraemic infections.

## Conclusions

The study found that nDNA outperformed other DAMPs regarding both sepsis detection and outcome prediction. Initial concentrations of nDNA and 16S rDNA were correlated to each other and were independently correlated with sepsis, underscoring their potential for severity assessment. nDNA was also correlated with negative outcome. Further research is needed to establish the diagnostic and prognostic properties of nDNA and to determine if elevated nDNA is merely a result of severe illness or if it is involved in sepsis pathogenesis, potentially making it a therapeutic target.

## Supplementary Information

Supplementary Information.

## Data Availability

16S rDNA-data has been published in Ziegler et al. PLoS One 2019;14(11):e0224656. The remaining datasets used and analysed during the current study are available from the corresponding author upon reasonable request.

## Abbreviations

DAMP: Damage-associated molecular pattern
PAMP: Pathogen-associated molecular pattern
nDNA: Nuclear DNA
mtDNA: Mitochondrial DNA
HSP90α: Heat shock protein 90 alpha
HMGB1: Extracellular high mobility group box 1
ICU: Intensive care unit
ROC: Receiver operating characteristic
PRR: Pattern recognition receptor
*S.** pneumoniae*: *Streptococcus pneumonia*
*S.** aureus*: *Staphylococcus aureus*
*E.** coli*: *Escherichia coli*
BC: Blood culture
CCI: Charlson comorbidity index
SOFA score: Sequential organ failure assessment score
mHLA-DR: Monocytic HLA-DR expression
