# Supplementary material for: Damage-associated molecular patterns in bacteraemic infection, including a comparative analysis with bacterial DNA, a pathogen-associated molecular pattern
Source: Sci Rep. 2024 Oct 8;14:23499. doi: 10.1038/s41598-024-74868-6 (PMC11461503; doi:10.1038/s41598-024-74868-6)
Supplement: Supplementary file 1 — Supplementary Information. [file 41598_2024_74868_MOESM1_ESM.pdf]

Damage-Associated Molecular Patterns in bacteraemic infection, including a comparative analysis with bacterial DNA, a Pathogen-Associated Molecular Pattern

Alpkvist et al.

Supplementary Information

Supplementary Table S1. Patient characteristics of the study population of patients with bacteraemic infection. Data are presented as numbers (percentages) for categorical variables and as median values (ranges) for continuous variables.

**Characteristics**	**All patients n=83**	**Patients with analysed bacterial DNA n=63**
Demographic data		
Female gender, n (%)	39 (47)	28 (44)
Age, median (range) years	72 (24-93)	71 (29-92)
Comorbidities		
Ischemic heart disease, n (%)	19 (23)	15 (24)
Cronic heart failure, n (%)	12 (14)	9 (14)
Cronic lung disease, n (%)	6 (7)	6 (10)
Cronic renal failure, n (%)	6 (7)	4 (6)
Liver disease, n (%)	0 (0)	0 (0)
Cerebrovascular disease, n (%)	15 (18)	9 (14)
Dementia, n (%)	3 (4)	2 (3)
Diabetes mellitus, n (%)	18 (22)	12 (19)
Active malignancy, n (%)	9 (11)	6 (10)
Immuno-suppressive treatment[a], n (%)	8 (10)	4 (6)
Comorbidity index		
Charlson comorbidity index, median (range)	1 (0-8)	1 (0-8)
Disease severity		
Admission SOFA score, media (range)	1 (0-7)	2 (0-7)
Sepsis at admission (SOFA score increase ≥2), n (%)	41 (49)	37 (59)
Hospitalization length, median (range) days	8 (2-120)	8 (2-120)
Intensive care unit admission, n (%)	12 (14)	11 (17)
Mortality ≤60 days after admission, n (%)	7 (8)	7 (11)
Negative outcome (ICU admission and/or mortality ≤60 days), n (%)	17 (20)	16 (25)

[a] Methotrexate, chemotherapeutics, or cortisol dosing equivalent to ≥20 mg Prednisolone.

Supplementary Table S2. Patient baseline characteristics and their correlation with plasma nDNA log10 concentration on day 1-2. Linear regression analysis in 83 patients with culture-proven bacteraemic infection, univariate analysis.

**Patient baseline characteristic**	**Coefficient beta**	**SE**	**P-value**
Male gender	-0.003	0.094	0.97
Age	-0.002	0.003	0.98
Charlson comorbidity index	0.087	0.022	0.47

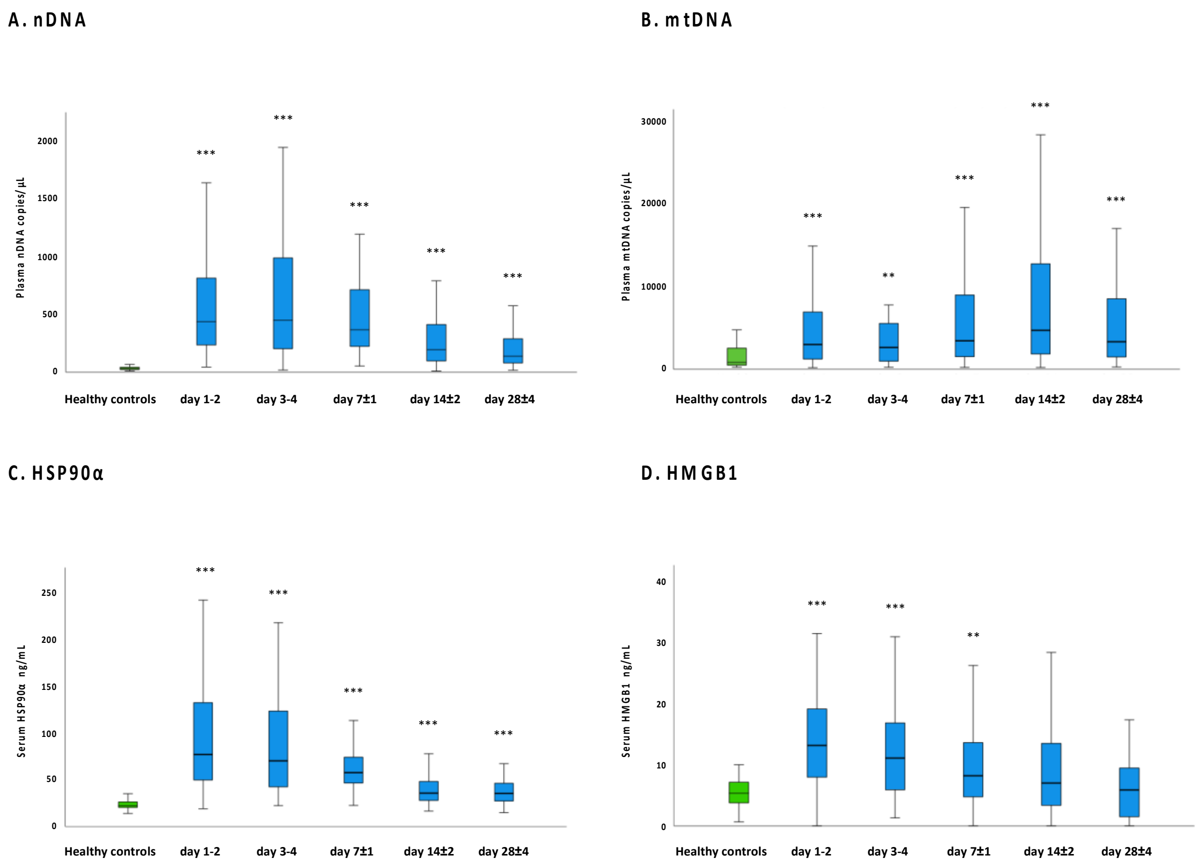

Supplementary Fig. S1A-D. DAMP concentrations over time in patients with bacteraemic infection, vs. in healthy controls. *=p<0.05, **=p<0.01 and ***=p<0.001. A small number of outliers were omitted from the visual presentations to allow for the use of a linear scale while maintaining readability. However, all outliers were included in all statistical analyses.

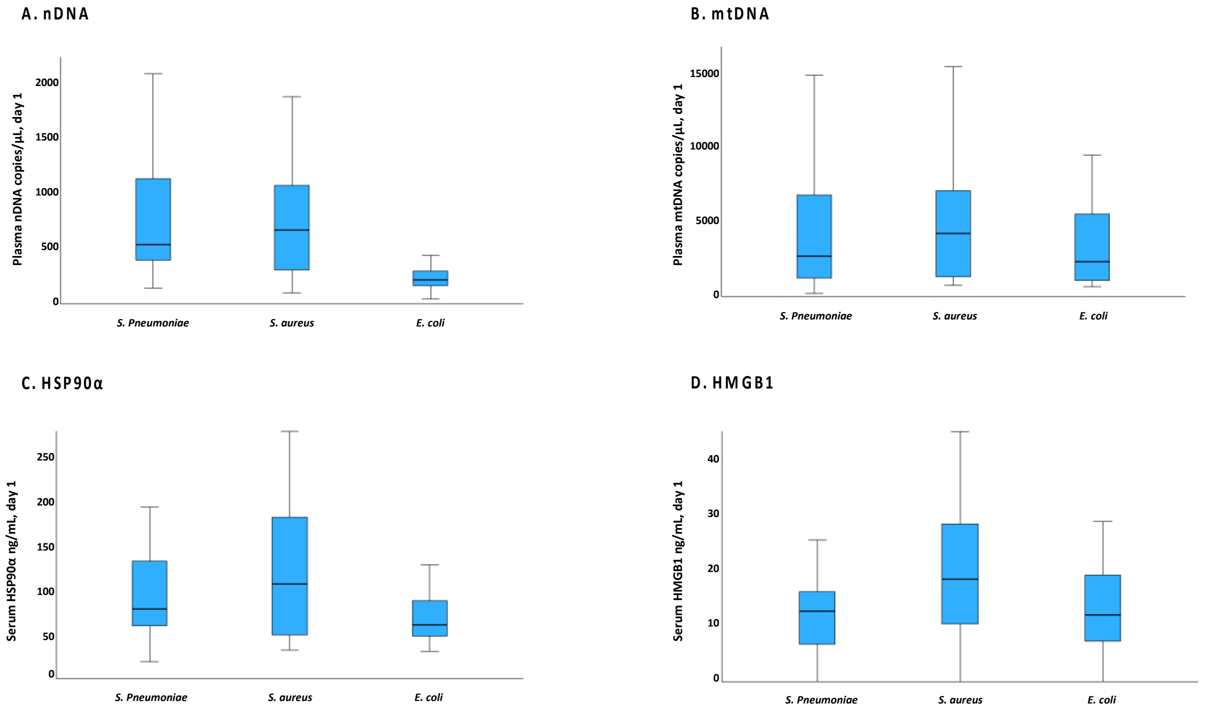

Supplementary Fig. S2A-D. DAMP concentrations on day 1-2, comparing patients with different etiologies. nDNA concentrations (A) were significantly higher in patients with *Streptococcus pneumoniae* bacteraemia (median 534, interquartile range 373-1157, copies/µL) or *Staphylococcus aureus* bacteraemia (median 661, interquartile range 301-1068, copies/µL) than in those with *Escherichia coli* bacteraemia (median 215, interquartile range 149-316, copies/µL), $p<0.001$ for both comparisons, Kuskal Wallis test. No significant difference was not seen for mtDNA, HSP90α or HMGB1 concentrations (B, C, D). A small number of outliers were omitted from the visual presentations to allow for the use of a linear scale while maintaining readability. However, all outliers were included in all statistical analyses.

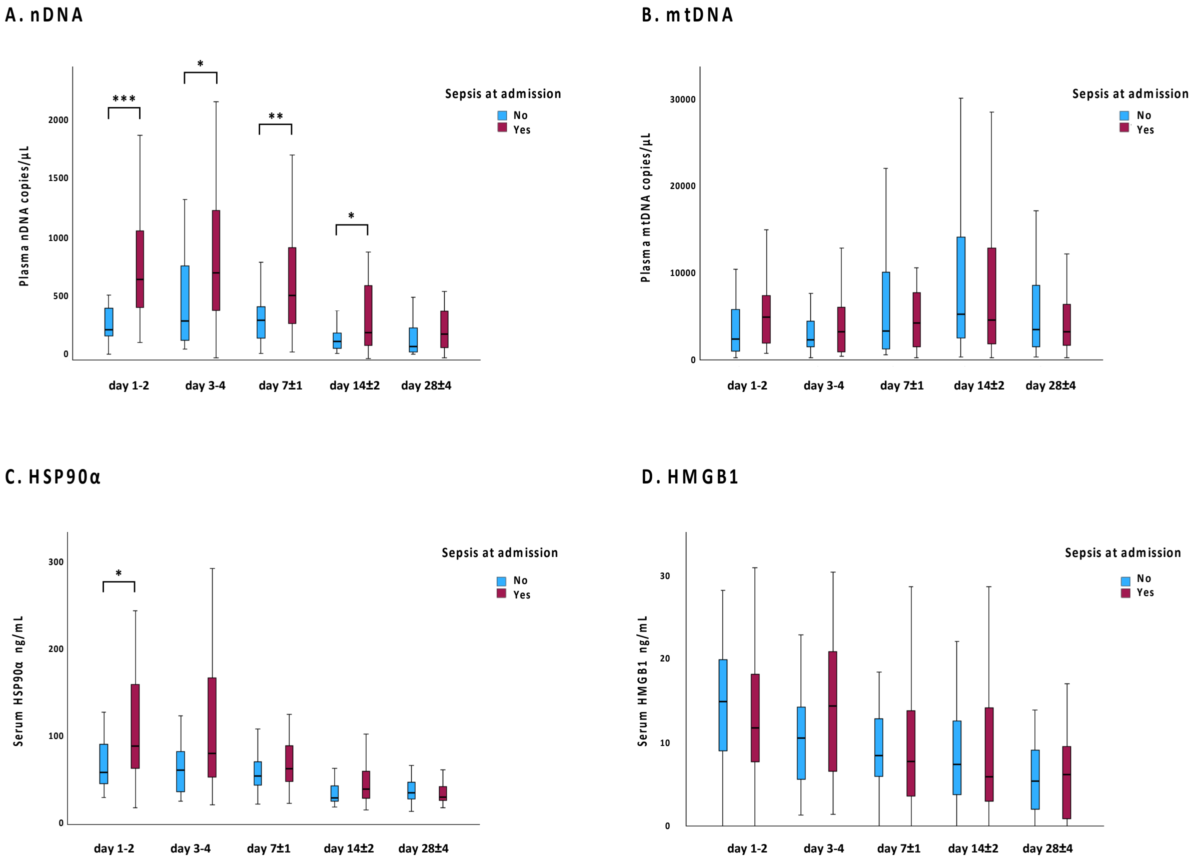

Supplementary Fig. S3A-D. DAMP concentrations on day 1 to day 28 in patients with sepsis (SOFA score ≥2) vs. patients without sepsis (SOFA score <2) at admission. *=p<0.05, **=p<0.01 and ***=p<0.001. A small number of outliers were omitted from the visual presentations to allow for the use of a linear scale while maintaining readability. However, all outliers were included in all statistical analyses.
